# ChatGPT in pharmacy practice: a cross-sectional exploration of Jordanian pharmacists' perception, practice, and concerns

**DOI:** 10.1186/s40545-023-00624-2

**Published:** 2023-10-03

**Authors:** Khawla Abu Hammour, Hamza Alhamad, Fahmi Y. Al-Ashwal, Abdulsalam Halboup, Rana Abu Farha, Adnan Abu Hammour

**Affiliations:** 1 Department of Clinical Pharmacy and Biopharmaceutics, Faculty of Pharmacy, University of Jordan, Amman, Jordan; 2 Department of Clinical Pharmacy, Faculty of Pharmacy, Zarqa University, Zarqa, Jordan; 3 Department of Clinical Pharmacy, College of Pharmacy, Al-Ayen University, Thi-Qar, Iraq; 4 Department of Clinical Pharmacy and Pharmacy Practice, Faculty of Pharmacy, University of Science and Technology, Sana’a, Yemen; 5 Discipline of Clinical Pharmacy, School of Pharmaceutical Sciences, University Sains Malaysia, Gelugor, Pulau Pinang Malaysia; 6 Clinical Pharmacy and Therapeutics Department, Faculty of Pharmacy, Applied Science Private University, P.O. Box 11937, Amman, Jordan; 7 Medrise Medical Center, Dubai Healthcare City, Dubai, United Arab Emirates

**Keywords:** Artificial intelligence, Pharmacy practice, ChatGPT

## Abstract

**Objectives:**

The purpose of this study is to find out how much pharmacists know and have used ChatGPT in their practice. We investigated the advantages and disadvantages of utilizing ChatGPT in a pharmacy context, the amount of training necessary to use it proficiently, and the influence on patient care using a survey.

**Methods:**

This cross-sectional study was carried out between May and June 2023 to assess the potential and problems that pharmacists observed while integrating chatbots powered by AI (ChatGPT) in pharmacy practice. The correlation between perceived benefits and concerns was evaluated using Spearman's rho correlation due to the data's non-normal distribution.Any pharmacists licensed by the Jordanian Pharmacists Association were included in the study. A convenient sampling technique was used to choose the participants, and the study questionnaire was distributed utilizing an online medium (Facebook and WhatsApp). Anyone who expressed interest in taking part was given a link to the study's instructions so they may read them before giving their electronic consent and accessing the survey.

**Results:**

The potential advantages of ChatGPT in the pharmacy practice were widely acknowledged by the participants. The majority of participants (69.9%) concurred that educational material about pharmacy items or therapeutic areas can be provided using ChatGPT, with 66.9% of respondents believing that ChatGPT is a machine learning algorithm. Concerns about the accuracy of AI-generated responses were also prevalent. More than half of the participants (55.7%) raised the possibility that AI systems such as ChatGPT could pick up on and replicate prejudices and discriminatory patterns from the data they were trained on. Analysis shows a statistically significant positive link, albeit a minor one, between the perceived advantages of ChatGPT and its drawbacks (*r* = 0.255, *p* < 0.001). However, concerns were strongly correlated with knowledge of ChatGPT. In contrast to those who were either unsure or had not heard of ChatGPT (64.2%), individuals who had heard of it were more likely to have strong concerns (79.8%) (*p* = 0.002). Finally, the results show a statistically significant association between the frequency of ChatGPT use and positive perceptions of the tool (*p* < 0.001).

**Conclusions:**

Although ChatGPT has shown promise in health and pharmaceutical practice, its application should be rigorously regulated by evidence-based law. According to the study's findings, pharmacists support the use of ChatGPT in pharmacy practice but have concerns about its use due to ethical reasons, legal problems, privacy concerns, worries about the accuracy of the data generated, data learning, and bias risk.

## Introduction

The multidisciplinary field of computer science and linguistics known as artificial intelligence (AI) aims to build robots capable of carrying out tasks that would typically need human intelligence [1]. These responsibilities include the capacity for learning, adaption, rationalization, comprehension, fathoming abstract concepts, and responsiveness to intricate human traits, such as attention, emotion, creativity, etc. [2]. The Dartmouth Summer Research Project on AI, which began in the middle of the twentieth century, is where the history of AI as a scientific field may be explored [3]. Following this, machine learning (ML) algorithms were created, enabling the creation of predictions or decisions based on the patterns seen in sizable data sets [4]. Genetic algorithms were subsequently developed using evolutionary principles to identify the best solutions to complicated problems, neural networks, and other cutting-edge methodologies [5].

Chatbots, powered by AI, have become increasingly popular in recent years as a tool to improve patient care and medication management. One of the most promising chatbot models is ChatGPT, a conversational agent built using the GPT (Generative Pre-trained Transformer) language model [6]. ChatGPT is designed to assist patients in answering health-related questions and providing personalized advice. However, the success of this technology depends on the knowledge and experience of the pharmacists who use it [7, 8]. ChatGPT has attracted a range of reactions from the academic and scientific communities, reflecting the long debate about the advantages and disadvantages of cutting-edge AI technologies [9–11]. On one hand, ChatGPT, along with other Large Language Models (LLMs), can be useful for conversational and writing jobs, helping to improve the effectiveness and correctness of the required output [12]. On the other hand, questions have been raised about potential bias based on the data sets used in ChatGPT training, which may limit its capabilities and lead to factual inaccuracies but alarmingly appear to be academically reasonable (a phenomenon known as hallucination) [12]. In addition, security issues and the potential for breaches with the dissemination of misleading data through LLMs should be considered [12]. This research aims to investigate pharmacists' current knowledge and experience regarding using ChatGPT in their practice. Through a survey, we explored the benefits and drawbacks of using ChatGPT in a pharmacy setting, the level of training required to use it effectively, and the impact on patient care. This research will provide valuable insights into the pharmacists’ perceived challenges and opportunities of implementing AI-powered chatbots in pharmacy practice, and inform strategies to optimize their use.

## Methods

### Study design and setting

This cross-sectional study was conducted between May and June 2023 to evaluate pharmacists’ perceived challenges and opportunities to implement AI-powered chatbots (ChatGPT) in pharmacy practice. The study included pharmacists licensed by the Jordanian Pharmacists Association. A convenient sampling method was used to select the participants by distributing the study questionnaire using an online platform (Facebook and WhatsApp). Those who expressed interest in participating were provided with a link to view the study's instructions and to provide their electronic consent before proceeding to the survey.

### Survey development

The initial study questionnaire was generated and evaluated for content and face validity by two survey experts. Minor modifications were made based on the notes and feedback provided. The comment included phrases such, "The survey is concise; kindly review questions, which could be merged to reduce the total number of questions to ensure a higher completion rate." as an example. The final version of the questionnaire consisted of four parts. The first part comprised demographic characteristics of the study population. The second part included statements to assess pharmacists' perceived benefits of ChatGPT incorporation in pharmacy practice. The third section included statements to evaluate pharmacists’ concerns related to ChatGPT incorporation in pharmacy practice. The last part evaluated pharmacists’ practice in using ChatGPT.

A 5-point Likert scale ranging from strongly disagree to strongly agree was utilized to evaluate the pharmacists’ perceived benefits and concerns, with scores ranging from 1 to 5, respectively. Perceived benefits and concerns were divided into high and low categories using the 80% Bloom's cutoff point as the threshold (1). For perceived benefits, scores varied between 18 and 54, with scores from 44 to 54 (≥ 80%) considered good perceived benefits. For concerns, the scores ranged from 5 to 15, with 12 to 15 (≥ 80%) denoting high concern levels.

### Sample size determination

The sample size was estimated using Raosoft ® sample size calculator for online survey using the following formula: *n* = *P* × (1 − *P*) × *z*^2^/*d*^2^. With a margin of error (*d*) of 10%, a confidence level of 95%, and a response distribution (*P*) of 50%, the minimum recommended sample size was 96 participants.

### Ethical considerations

Ethical approval for the study was granted by the Institutional Review Board at the Jordan University Hospital (Approval number: 10/2023/16283). The current study was conducted following the Declaration of Helsinki of the World Medical Association.

### Statistical analysis

The data analysis was conducted using SPSS software, version 25 (IBM Corp., Armonk, NY, USA). A mix of inferential and descriptive statistical methods was applied depending on the nature of the data. Frequency and percentages were computed for categorical variables. The data normality was examined using the Kolmogorov–Smirnov test and histogram visualizations, revealing a non-normal distribution. The test yielded a significant result (*p* < 0.05), affirming this non-normal distribution.

The correlation between perceived benefits and concerns was evaluated using Spearman's rho correlation due to the data's non-normal distribution. 

Factors associated with poor perception and high concerns were assessed using the Pearson Chi-square test. It was used to investigate associations between participants' demographic data and high perceived benefits and concerns levels. The same test examined the relationship between ChatGPT practices and perceptions and concerns. A *p* value of less than 0.05 was deemed to signify statistically significant results.

## Results

### Sociodemographic characteristics

The demographic profile of the participants in the study, as shown in Table 1, indicated that the majority were in the age bracket of 20–30 years, accounting for 298 (83.2%) of the participants. Female participants outnumbered males, with 60.7% (*n* = 212) compared to 39.3% (*n* = 141), respectively. Regarding educational background, the majority of participants held a BSc degree (88.5%, *n* = 317). When examining the participants' profession, the largest group worked in community and hospital pharmacies (38.4%, *n* = 138). Participants working in academic and research fields comprised 9.7% (*n* = 35) of the sample, and those in drug manufacturing companies represented 18.1% (*n* = 65).

**Table 1:** Participant's sociodemographic data (*n* = 359)

Variable	Category	Count (%)
Age (year)	20–30	298 (83.2)
	31–40	49 (13.7)
	41–51	11 (3.1)
Gender	Male	141 (39.3)
	Female	218 (60.7)
Degree	BSc	317 (88.5)
	Master	31 (8.7)
	PhD	10 (2.8)
Profession	Academic and research	35 (9.7)
	Community and hospital pharmacies	138 (38.4)
	Drug manufacturing company	65 (18.1)
	Others (management, health insurance and regulatory, medical representative, business, marketing)	121 (33.7)
A prior understanding of technology for artificial intelligence or natural language processing	Significant experience in pharmacy practice	32 (8.9)
	Some in pharmacy practice	100 (27.9)
	Some, but not in pharmacy practice	174 (48.5)
	None	53 (14.8)
Proficiency with digital technology and computers (grade yourself)	Poor	11 (3.1)
	Fair	43 (12.0)
	Good	130 (36.2)
	Very good	122 (34.0)
	Excellent	53 (14.8)
Have you heard of ChatGPT?	Yes	253 (70.5)
	No/Not sure	106 (29.5)

The level of understanding of technology for AI or natural language processing varied among participants. A small percentage (8.9%, *n* = 32) had significant experience in pharmacy practice, whereas less than a third (27.9%, *n* = 100) had some experience in the same area. The largest group had some technological understanding but not specifically in pharmacy practice (48.5%, *n* = 174), and only 14.8% (*n* = 53) of the participants reported no prior understanding.

More than a third of participants reported proficiency with digital technology and computers as 'Good' (36.2%, *n* = 130), followed closely by those who rated their proficiency as 'Very Good' (34.0%, *n* = 122). A minority of participants rated their proficiency as Fair (12.0%, *n* = 43) or 'Poor' (3.1%, *n* = 11). Concerning ChatGPT, a significant proportion of participants (70.5%, *n* = 253) reported having heard of ChatGPT.

### Perception toward the use of ChatGPT benefits in pharmacy practice

An overview of the perception responses in Table 2 suggests that many participants recognized the potential benefits of using ChatGPT in the pharmaceutical sector. More specifically, the survey's highest concurrence (77.2%) came from the statement asserting that pharmacists could greatly benefit from using ChatGPT. Another area with a substantial agreement (69.1%) was the use of ChatGPT for content creation, which suggests that participants recognize the potential of AI for generating ideas for marketing materials, such as blog posts and social media pharmacy posts. Similarly, over two-thirds of participants (69.9%) agreed that ChatGPT can be used to provide educational content related to pharmacy products or therapeutic areas.

**Table 2:** Participants’ benefits perceptions about ChatGPT (*n* = 359)

Statement	Agree *N* (%)	Neutral *N* (%)	Disagree *N* (%)
Pharmacists can benefit from using ChatGPT	277 (77.2)	75 (20.9)	7 (1.9)
ChatGPT can provide accurate information regarding medicine	169 (47.1)	142 (39.6)	48 (13.4)
ChatGPT can assist in product training by providing accurate and up-to-date information	197 (54.9)	128 (35.7)	34 (9.5)
ChatGPT can be used to provide 24/7 customer support to patients, healthcare providers, and other stakeholders	186 (51.8)	122 (34)	51 (14.2)
ChatGPT can be used to match patients with clinical trials based on their medical history and other criteria	186 (51.8)	128 (35.7)	45 (12.5)
ChatGPT can be used to provide medical education and training to healthcare professionals	191 (53.2)	120 (33.4)	48 (13.4)
ChatGPT can be used to analyze patient data and provide personalized treatment recommendations based on their unique medical history and genetic profile	173 (48.2)	132 (36.8)	54 (15)
ChatGPT can assist in content creation by generating ideas for blog posts, social media posts, and other marketing materials	248 (69.1)	98 (27.3)	13 (3.6)
ChatGPT can be used to provide educational content related to your products or therapeutic areas	240 (66.9)	102 (28.4)	17 (4.7)
ChatGPT can assist sales representatives by answering common questions about the products they are promoting	227 (63.2)	114 (31.8)	18 (5)
ChatGPT can also be used to create role-playing scenarios, where trainees can practice their sales pitch or communication skills in a safe and controlled environment	226 (63)	118 (32.9)	15 (4.2)
ChatGPT can be programmed to identify quality control issues during the manufacturing process	193 (53.8)	134 (37.3)	32 (8.9)
ChatGPT can analyze manufacturing data to identify areas, where the manufacturing process can be optimized to improve efficiency or quality	210 (58.5)	123 (34.3)	26 (7.2)
ChatGPT can be used to monitor equipment performance and detect potential issues before they become major problems	173 (48.2)	152 (42.3)	34 (9.5)
ChatGPT can assist in research and development by analyzing data and identifying trends that can lead to the development of new and improved pharmaceutical products	224 (62.4)	110 (30.6)	25 (7)
ChatGPT can be used to provide training on regulatory requirements and best practices related to compliance	206 (57.4)	130 (36.2)	23 (6.4)
ChatGPT can monitor and analyze data to identify potential compliance issues, such as deviations from established manufacturing processes or regulatory requirements	193 (53.8)	132 (36.8)	34 (9.5)
ChatGPT can help pharmaceutical companies manage compliance risks by identifying potential compliance issues before they become major problems	195 (54.3)	138 (38.4)	26 (7.2)

On the other hand, less than half of the participants (47.1%) agreed that ChatGPT can provide accurate information regarding medicine. In addition, the use of ChatGPT to analyze patient data and provide personalized treatment recommendations based on the patient's unique medical history and genetic profile met with the most disagreement, with 51.8% of participants expressing dissent or being neutral. Although this topic received the highest disagreement and neutrality, it is worth noting that it still received 48.2% of agreement.

Further examination of the results brings to light the participants' perspectives on using ChatGPT as a monitoring tool for equipment performance, with the potential to detect issues before they become significant problems. Close to half of the participants (48.2%) agreed with this proposition. However, a considerable segment (42.3%) expressed neutrality.

### Concerns about the use of ChatGPT in pharmacy practice

**Fig. 1:**
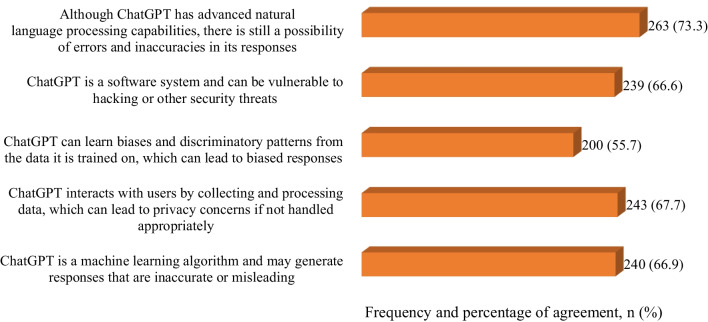
Agreement of the participants to the concern statements about ChatGPT

While the previous analysis showed a generally positive attitude toward ChatGPT's applications in the pharmaceutical sector, some significant concerns were also highlighted (Fig. 1). Despite its advanced natural language processing capabilities, nearly three-quarters of the participants (73.3%) expressed apprehensions about possible errors and inaccuracies in ChatGPT's responses. The issues of privacy and data security were also underscored, with 67.7% worrying about potential privacy concerns due to data collection and processing and 66.6% pointing out the vulnerability of software systems such as ChatGPT to hacking or other security threats. Concerns over the accuracy of AI-generated responses were also prominent, with 66.9% agreeing that ChatGPT might generate inaccurate or misleading responses as a machine learning algorithm. In addition, over half of the participants (55.7%) brought attention to the potential for AI systems such as ChatGPT to learn and reproduce biases and discriminatory patterns from the data they are trained on.

### The correlation between recognized benefits of ChatGPT and identified concerns

Table 3 illustrates the correlation between perceived benefits and concerns. The Spearman's rho correlation coefficient is 0.255 (*p* < 0.001), with a significance level of *p* < 0.001. This indicates a statistically significant positive correlation, albeit weak, between the perceived benefits of ChatGPT and its concerns that is statistically significant.

**Table 3:** Correlation between perceived benefits and concerns

			Perceived benefits	Perceived concerns
Spearman's rho	Perceived benefits	Correlation coefficient	1.000	0.255
		*p* value		< 0.001*
	Perceived concerns	Correlation coefficient	0.255	1.000
		*p* value	< 0.001*	

*Correlation is significant at the 0.05 significance level

### Factors associated with poor perception and high concerns

Table 4 shows that age, gender, degree level, profession, prior understanding of technology for AI, and proficiency with digital technology were not significantly associated with poor or good perception or with high or low concerns (*p* > 0.05). However, the awareness about ChatGPT was significantly associated with concerns. For instance, participants who had heard of ChatGPT were more likely to have high concerns (79.8%) as compared to those who were either unsure or had not heard about it (64.2%) (*p* = 0.002).

**Table 4:** Association between participants' sociodemographic data and their perception and concerns about ChatGPT (n = 359)

Parameters		Perception		Chi-square	*p* value	Concerns		Chi-square	*p* value
		Poor *n* = 145, *n* (%)	Good *n* = 214, *n* (%)			Low *n* = 89, *n* (%)	High *n* = 270, *n* (%)		
Age	20–30	116 (38.3)	138 (61.2)	2.689	0.261	71 (23.7)	228 (76.3)	1.102	0.576
	31–40	25 (51)	24 (49)			15 (30.6)	34 (69.4)		
	41–51	4 (36.4)	7 (63.6)			3 (27.3)	8 (72.7)		
Gender	Male	53 (37.6)	88 (62.4)	0.757	0.384	39 (27.7)	102 (72.3)	1.025	0.311
	Female	92 (42.2)	126 (57.8)			50 (22.9)	168 (77.1)		
Degree	Undergraduate	133 (41.8)	185 (58.2)	2.378	0.123	76 (23.9)	242 (76.1)	1.187	0.276
	Postgraduate	12 (29.3)	29 (70.7)			13 (31.7)	28 (68.3)		
Professional	Academic and research	14 (40)	21 (60)	2.036	0.565	6 (17.1)	29 (82.9)	2.069	0.558
	Community and hospital pharmacies	52 (37.7)	86 (62.3)			38 (27.5)	100 (72.5)		
	Drug manufacturing company	24 (36.9)	41 (63.1)			14 (21.5)	51 (78.5)		
	Others^∞^	55 (45.5)	66 (54.5)			31 (25.6)	90 (74.4)		
A prior understanding of technology for artificial intelligence or natural language processing	Significant experience in pharmacy practice	12 (37.5)	20 (62.5)	1.242	0.743	6 (18.7)	26 (81.3)	3.500	0.321
	Some in pharmacy practice	39 (39)	61 (61)			28 (28)	72 (72)		
	Some, but not in pharmacy practice	69 (39.7)	105 (60.3)			38 (21.8)	136 (78.2)		
	None	25 (47.2)	28 (52.8)			17 (32.1)	36 (67.9)		
Proficiency with digital technology and computers	Poor	3 (27.3)	8 (72.7)	1.067	0.900	2 (18.2)	9 (81.8)	1.798	0.773
	Fair	19 (44.2)	24 (55.8)			12 (27.9)	31 (72.1)		
	Good	53 (40.8)	77 (59.2)			35 (26.9)	95 (73.1)		
	Very good	49 (40.2)	73 (59.8)			30 (24.6)	92 (75.4)		
	Excellent	21 (39.6)	32 (60.4)			10 (18.9)	43 (81.1)		
Have you heard of ChatGPT?	Yes	105 (41.5)	148 (58.5)	0.440	0.507	51 (20.2)	202 (79.8)	9.864	0.002*
	No/Not sure	40 (37.7)	66 (62.3)			38 (35.8)	68 (64.2)		

^*^Significant at the 0.05 significance level, ∞ Others involve: management, health insurance and regulatory, medical representative, business, and marketing

### Practices of pharmacists in using ChatGPT

Table 5 shows the participant’s responses to using ChatGPT in pharmacy practice. The majority of the participants, approximately 49.9%, stated that they have never used ChatGPT in their pharmaceutical practice, while 29% reported using it rarely, i.e., once a month. The usage was occasional (2–3 times a month) for 13.6% of the participants, and only a small fraction, 7.5%, said they use it frequently, at least once a week. Regarding the recommendation of ChatGPT to other pharmacists, a majority of participants (61.6%) responded affirmatively. However, a significant portion, 30.6%, remained undecided, and a small percentage, 7.8%, expressed their disagreement.

**Table 5:** Participants’ response to the use of ChatGPT in pharmacy practice (*n* = 359)

Practice statement	Category	Frequency (%)
Current frequency of using ChatGPT in pharmaceutical areas	Frequently once a week or more	27 (7.5)
	Occasionally (2–3) times a month	49 (13.6)
	Rarely (once a month)	104 (29)
	Never	179 (49.9)
Would you recommend the use of ChatGPT to other pharmacists?	Yes	221 (61.6)
	Undecided	110 (30.6)
	No	28 (7.8)
Have you used the ChatGPT to check for Drug–drug interactions?	Yes	69 (19.2)
	No	260 (80.8)
Have you ever used the ChatGPT as a drug and disease information source (e.g., ask ChatGPT questions about particular conditions, treatments, medications, or lifestyle changes)?	Yes	104 (29.0)
	No	255 (71.0)
Have you ever used ChatGPT for medication reconciliation?	Yes	56 (15.6)
	No	303 (84.4)
Have you ever used ChatGPT to determine appropriate dosage regimens for patients?	Yes	58 (16.2)
	No	301 (83.8)
Have you ever used ChatGPT to identify or manage adverse drug reactions?	Yes	80 (22.3)
	No	279 (77.7)

When queried about the specific applications of ChatGPT in their practice, the responses varied. Checking for drug–drug interactions was performed only by 19.2% of the pharmacists. Similarly, less than a third of the participants (29%) have used ChatGPT as a drug and disease information source. Furthermore, only 15.6% reported using ChatGPT for medication reconciliation and 16.2% for determining appropriate dosage regimens for patients, with the majority not utilizing it for these purposes. Finally, a slightly higher proportion, 22.3%, used the AI tool to identify or manage adverse drug reactions.

### Usefulness of using ChatGPT compared to other standard resources

**Fig. 2:**
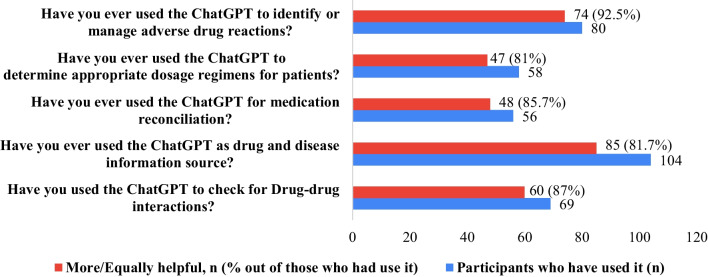
Usefulness of using ChatGPT compared to other standard resources

Figure 2 further delves into the perceived usefulness of ChatGPT among those who have employed it in their pharmaceutical practice, comparing it to standard resources. For those who utilized ChatGPT to check for drug–drug interactions, an overwhelming 87% found it to be more or equally helpful. Similarly, among the pharmacists who used ChatGPT as a drug and disease information source, approximately 81.7% reported it to be as useful as, or more beneficial than, conventional resources (e.g., UpToDate, Medscape, Lexicomp, therapy Textbooks, etc.).

For medication reconciliation, a task performed by 56 participants, a significant proportion (85.7%) found ChatGPT either equally useful or superior to human-made decisions. The utility of ChatGPT was also recognized in determining appropriate dosage regimens for patients, with 81% of the 58 participants who used it for this purpose acknowledging its value. Interestingly, the highest percentage of positive responses was noted for managing or identifying adverse drug reactions. Here, an impressive 92.5% of the 80 users reported that ChatGPT was as good as or better than traditional sources.

### Association between ChatGPT practice and participants’ perceptions and concerns

Table 6 displays the association between ChatGPT practice and participants’ perceptions and concerns. The data show a statistically significant association between the frequency of ChatGPT use and positive perceptions of the tool (*p* < 0.001). Those who used it frequently had a higher percentage (92.6%) of good perception compared to those who used it occasionally (67.3%), rarely (60.6%), or never (52%). In other words, the more frequently pharmacists used ChatGPT, the more favorably they viewed it. When considering the recommendation of ChatGPT to other pharmacists, those who would recommend it had a significantly higher percentage (72.4%) of good perception compared to those who would not recommend it (25%) (*p* = 0.001). For specific applications of ChatGPT in pharmacy practice, participants who used it to check for drug–drug interactions, as a drug and disease information source, for medication reconciliation, to determine appropriate dosage regimens for patients, or to identify or manage adverse drug reactions all had significantly a higher percentage of good perception compared to those who have not use it (*p* = 0.001).

**Table 6:** Association between the use of ChatGPT in pharmacy practice and perception and concerns

Parameters		Perception		Chi-square	*p* value	Concerns		Chi-square	*p* value
		Poor *N* = 145, *n* (%)	Good *N* = 214, *n* (%)			Low *N* = 89, *n* (%)	High *N* = 270, *n* (%)		
Frequency of ChatGPT use	Frequently	2 (7.4)	25 (92.6)	17.814	< 0.001*	5 (18.5)	22 (81.5)	7.152	0.067
	Occasionally	16 (32.7)	33 (67.3)			14 (28.6)	35 (71.4)		
	Rarely	41 (39.4)	63 (60.6)			17 (16.3)	87 (83.7)		
	Never	86 (48)	93 (52)			53 (29.6)	126 (70.4)		
Recommend ChatGPT to other pharmacists	Yes	61 (27.6)	160 (72.4)	41.964	< 0.001*	46 (20.8)	175 (79.2)	15.743	< 0.001*
	Undecided	63 (57.3)	47 (42.7)			41 (37.3)	69 (62.7)		
	No	21 (75)	7 (25)			2(7.1)	26 (92.9)		
Have you used the ChatGPT to check for Drug–drug interactions?	Yes	16 (23.2)	53(76.8)	10.498	0.001*	20 (29)	49 (71)	0.806	0.369
	No	129 (44.5)	161 (55.5)			69 (23.8)	221 (76.2)		
Have you ever used the ChatGPT as a drug and disease information source (e.g., ask ChatGPT questions about particular conditions, treatments, medications, or lifestyle changes)?	Yes	25 (24)	79 (76)	16.260	< 0.001*	25 (24)	79 (76)	0.044	0.833
	No	120 (47.1)	135 (52.9)			64 (25.1)	191 (74.9)		
Have you ever used ChatGPT for medication reconciliation?	Yes	11 (19.6)	45 (80.4)	11.862	0.001*	10 (17.9)	46 (82.1)	1.711	0.191
	No	134 (44.2)	169 (55.8)			79 (26.1)	224 (73.9)		
Have you ever used ChatGPT to determine appropriate dosage regimens for patients?	Yes	12 (20.7)	46 (79.3)	11.151	0.001*	15 (25.9)	43 (74.1)	0.043	0.868
	No	133 (44.2)	168 (55.8)			74 (24.6)	227 (75.4)		
Have you ever used ChatGPT to identify or manage adverse drug reactions?	Yes	16 (20)	64 (80)	17.775	< 0.001*	17 (21.3)	63 (78.8)	0.692	0.405
	No	129 (46.2)	150 (53.8)			72 (25.8)	207 (74.2)		

^*^Significant at the 0.05 significance level

The data paints a slightly different picture of the association between ChatGPT practices and concerns. Interestingly, the survey data showed that those who did not favor recommending ChatGPT had noticeably more concerns than those who would recommend it. This difference was statistically significant, with a high 92.9% of the non-recommenders expressing high concerns, compared to 79.2% among those advocating for the tool (*p* < 0.001).

## Discussion

Artificial Intelligence (AI) applications, including ChatGPT, would be considered a paradigm shift in healthcare academia and practice in the current and upcoming future [13–16]. With a growing number of studies highlighting healthcare providers' perceptions, concerns, and practices toward applying ChatGPT in providing patients health and pharmacy practice [14, 15, 17–19], this study contributes by exploring the Jordanian pharmacists’ perceptions, concerns, and practices toward using ChatGPTin pharmacy practice.

In this study, the majority of participants recognised the potential benefits of applying ChatGPT in the pharmaceutical sector, with more than two-thirds asserting that pharmacists would greatly benefit from using ChatGPT, especially in generating ideas related to pharmaceutical marketing, such as social media and blogs, and in providing educational content related to pharmacy products or therapeutic areas. This is consistent with results reported the great benefits of ChatGPT in healthcare education, research, practice, and marketing [13, 14, 20–25].

However, less than half of the participants agreed that ChatGPT would provide accurate medicine-related information. This was consistent with the report ensuring the accuracy and relevancy of ChatGPT responses [13, 26]. However, other reports contrasted with our findings, ensuring the risk of bias in having inaccurate information with possible serious medical consequences [17, 20–22, 24, 27–29]. In addition, the use of ChatGPT to analyze patient data and provide personalized treatment recommendations based on the patient's unique medical history and genetic profile met with the most disagreement, with 51.8% of participants expressing dissent or being neutral. Despite this high level of disagreement and neutrality, it is essential to note that about half of the participants still agreed. Nevertheless, using AI in personalized patient treatment appears to be a more contentious subject among participants [30, 31]. These results would indicate a generally positive perception of ChatGPT's potential role in the pharmaceutical industry, with a few areas needing further discourse and exploration.

Regarding using ChatGPT as a monitoring tool for equipment performance, with its potential ability to detect issues before they become apparent problems, half of the participants agreed on this, with more than one-third being neutral. This would indicate uncertainty or lack of information about the practical application of AI in equipment monitoring. The potential benefit of ChatGPT as a monitoring tool for equipment performance was reported in other studies [32, 33].

Considering the concerns about using ChatGPT in pharmacy, findings in this study showed that more than two-thirds of participants were apprehensive about possible errors and inaccuracies in ChatGPT's responses despite ChatGPT's advanced natural language processing capabilities. In addition, less than two-thirds of participants were worried about potential privacy concerns due to data collection and processing and pointing out the vulnerability of software systems such as ChatGPT to hacking or other security threats. Less than two-thirds of participants reported concerns about the accuracy of AI-generated responses, and more than half of participants notified the potential for ChatGPT as an AI system to learn and reproduce biases and discriminatory patterns from the data they are trained on. The potential errors, inaccuracies, and ethical and privacy concerns were also reported in the literature [13, 18, 20, 22, 27–29, 34, 35]. These concerns underscore the need for careful implementation, stringent security measures, and continuous monitoring of AI systems, such as ChatGPT. Despite the perceived benefits, it is clear that a significant portion of the participants maintains a degree of skepticism toward the use of AI in the pharmaceutical sector, emphasizing the need for more discourse, transparency, and user education [36].

Regarding the correlation between the recognized benefits of ChatGPT and identified concerns. Findings indicate a statistically significant positive correlation, albeit weak, between the perceived benefits of ChatGPT and its concerns. This could imply that those who recognize more benefits in ChatGPT also tend to have more concerns and may also reflect a heightened awareness and understanding of the technology, whereby those more familiar with ChatGPT can appreciate its potential benefits but are simultaneously aware of its limitations or potential issues. They may have more experience with AI, which allows them to perceive a wide range of benefits from using it, but their experiences may also have exposed them to potential problems, thus elevating their concern levels. However, due to the relatively weak correlation, it is important to note that many other factors might influence the relationship between perceived benefits and concerns about ChatGPT, and further research is required to explore these potential influences.

Regarding the factors associated with poor perceptions and high concerns, findings indicate that age, gender, degree level, profession, prior understanding of technology for AI, and proficiency with digital technology were not significantly associated with poor or good perception or with high or low concerns. However, the awareness about ChatGPT was significantly associated with concerns. For example, the majority of participants who had heard of ChatGPT were more likely to have high concerns compared to those who were either unsure or had not heard about it. A significant correlation was observed between having heard about ChatGPT and heightened concerns regarding its use in pharmacy practice. This correlation could be attributed to the spread of exaggerated misinformation about the AI tool. Given AI's rapidly evolving nature and increasing integration into various sectors, it is not uncommon for misconceptions and inaccuracies to circulate. For instance, those who have heard about ChatGPT but have not used it might receive misleading or inaccurate information that could amplify their concerns about its reliability, efficacy, or potential risks. This misinformation could come from various sources, such as unreliable online content, hearsay, or misinterpretations of the technology's capabilities and limitations. Therefore, providing accurate, precise, and comprehensive information about ChatGPT and its potential applications in pharmacy practice is essential to alleviate these concerns and correct any misconceptions.

Regarding the practices of pharmacists in using ChatGPT in pharmacy practice, the majority of the participants reported that they have never used ChatGPT in their pharmacy practice, with less than one-third rarely (i.e., once a month) using it. One study reported a higher percentage, where clinical pharmacists used ChatGPT in prescription review, patient medication education, adverse drug reaction (ADR) recognition, ADR causality assessment, and drug counseling [37]. The ChatGPT usage was occasional (2–3 times a month) for 13.6% of the participants, and only a small fraction, 7.5%, reported frequent (i.e., at least once a week) use. In addition, more than half of the participants recommended ChatGPT to other pharmacists.

Participants' responses varied regarding the specific applications of ChatGPT in pharmacists’ practice. For example, less than a quarter of participants use ChatGPT to check for drug–drug interactions, for medication reconciliation, and to determine appropriate dosage regimens for patients, with the majority not utilizing it for these purposes. In addition, less than one-third used ChatGPT as a drug and disease information source to identify or manage adverse drug reactions. These results provide an overview of the current usage and perception of ChatGPT in the pharmaceutical field, indicating room for increased awareness and potential applications. One study in China reported that clinical pharmacists used ChatGPT in prescription review, patient medication education, adverse drug reaction (ADR) recognition, ADR causality assessment, and drug counseling [37].

Considering the perceived usefulness of ChatGPT in pharmacy practice compared to other standard resources. For those who used ChatGPT to check for drug–drug interactions, as a drug and disease information source, and for medication reconciliation, the majority of the participants found ChatGPT to be more or equally helpful and to be as useful as, or more beneficial than, conventional resources (e.g., UpToDate, Medscape, Lexicomp, Therapy Textbooks, etc.), and either equally useful or superior to human-made decisions, respectively. In addition, the majority of participants who used ChatGPT in determining appropriate dosage regimens for patients acknowledged its value, and the highest percentages of positive responses were noted for managing or identifying ADRs compared to traditional sources. These findings would indicate that despite the varying usage frequency in different pharmaceutical tasks, pharmacists who have used ChatGPT generally find it a valuable tool, often on par with or surpassing standard resources in terms of usefulness. However, it is essential to clarify that ChatGPT does not yet replace the pharmacist's clinical decision; it is a more useful tool that helps pharmacists provide timely pharmacy practice.

Regarding the association between ChatGPT practice and participants’ perceptions and concerns, findings showed a statistically significant association between the frequency of ChatGPT use and positive perceptions. In other words, the more frequently pharmacists used ChatGPT, the more favorably they viewed it. However, a slightly different picture of the association between ChatGPT practices and concerns with results showed that those who did not favor recommending ChatGPT had noticeably more concerns than those who would recommend it. This highlights that although ChatGPT's value is broadly recognized, significant reservations still need to be addressed, particularly among those less enthusiastic or less familiarised with the tool.

Findings from this study should be considered carefully in light of some limitations. The questionnaire was distributed online; therefore, data were collected only from pharmacists who use the Internet and other social media platforms were able to participate, and all information in this study was obtained through the self-report method, leading to a risk of social desirability bias or recall bias. In addition, a convenience sampling method was used, which may not represent the whole Jordanian pharmacists.

Findings from this study showed that Jordanian pharmacists positively endorse the benefit and the bright future of using ChatGPT as a tool compared to the available standard resources. However, it is important to clarify that limitations and concerns, such as ethical considerations, legal and privacy issues, the accuracy of the generated data, data learning, and risk of bias must be all addressed before widely implementing ChatGPT in pharmaceutical and healthcare. Huge volumes of medical data may be accessed through ChatGPT, which can answer clinical inquiries in real time with accuracy, promote patient involvement, and lighten the strain of medical professionals. However, as it has access to a lot of private health information, it can raise privacy issues. In addition, it might not always give accurate answers, especially in the case of difficult medical inquiries, and it is only as good as the data it was trained on. The model could reinforce a bias if the training set of data is skewed [38].

The results of this study would help healthcare providers and policymakers in Jordan and comparable global countries gain more insight about using ChatGPT in pharmacy practice, thus allowing them to react by defining the barriers and facilitators to its possible comprehensive implementation in the future, if any.

## Conclusion

The implementation of ChatGPT in health and pharmacy practice has been promising, but evidence-based legislation should carefully guide its use. The findings of this study showed that pharmacists have a positive endorsement of the use of ChatGPT in pharmacy practice but have concerns related to ethical considerations, legal and privacy issues, the accuracy of the generated data, data learning, and risk of bias. Future studies, including other healthcare providers, should be conducted, and more extensive studies should explore facilitators and barriers to implementing ChatGPT in health and pharmacy practice.

## Data Availability

The data that support the findings of this study are available from the corresponding author upon reasonable request.
