# Correction: ChatGPT in pharmacy practice: a cross-sectional exploration of Jordanian pharmacists’ perception, practice, and concerns

**DOI:** 10.1186/s40545-023-00640-2

**Published:** 2023-10-30

**Authors:** Khawla Abu Hammour, Hamza Alhamad, Fahmi Y. Al-Ashwal, Abdulsalam Halboup, Rana Abu Farha, Adnan Abu Hammour

**Affiliations:** 1 Department of Clinical Pharmacy and Biopharmaceutics, Faculty of Pharmacy, University of Jordan, Amman, Jordan; 2 Department of Clinical Pharmacy, Faculty of Pharmacy, Zarqa University, Zarqa, Jordan; 3 Department of Clinical Pharmacy, College of Pharmacy, Al-Ayen University, Thi-Qar, Iraq; 4 Department of Clinical Pharmacy and Pharmacy Practice, Faculty of Pharmacy, University of Science and Technology, Sana’a, Yemen; 5 Discipline of Clinical Pharmacy, School of Pharmaceutical Sciences, University Sains Malaysia, Gelugor, Pulau Pinang Malaysia; 6 Clinical Pharmacy and Therapeutics Department, Faculty of Pharmacy, Applied Science Private University, P.O. Box 11937, Amman, Jordan; 7 Medrise Medical Center, Dubai Healthcare City, Dubai, United Arab Emirates

**Correction: Journal of Pharmaceutical Policy and Practice (2023) 16:115** **https://doi.org/10.1186/s40545-023-00624-2**

Following publication of the original article [1], we have been notified of an error in Hamza Alhamad’s affiliation.

Originally published affiliation: Department of Clinical Pharmacy, Faculty of Pharmacy, Jordan University of Science and Technology, Irbid, Jordan.

Corrected affiliation: Department of Clinical Pharmacy, Faculty of Pharmacy, Zarqa University, Zarqa, Jordan.

The original article has been corrected.
